# Evaluation of Efficacy of Radioimmunotherapy with ^90^Y-Labeled Fully Human Anti-Transferrin Receptor Monoclonal Antibody in Pancreatic Cancer Mouse Models

**DOI:** 10.1371/journal.pone.0123761

**Published:** 2015-04-20

**Authors:** Aya Sugyo, Atsushi B. Tsuji, Hitomi Sudo, Maki Okada, Mitsuru Koizumi, Hirokazu Satoh, Gene Kurosawa, Yoshikazu Kurosawa, Tsuneo Saga

**Affiliations:** 1 Diagnostic Imaging Program, Molecular Imaging Center, National Institute of Radiological Sciences, Inage-ku, Chiba, Japan; 2 Molecular Probe Program, Molecular Imaging Center, National Institute of Radiological Sciences, Inage-ku, Chiba, Japan; 3 Department of Nuclear Medicine, Cancer Institute Hospital, Koto-ku, Tokyo, Japan; 4 Reserch and Development Division, Perseus Proteomics Inc., Meguro-ku, Tokyo, Japan; 5 Innovation Center for Advanced Medicine, Fujita Health University School of Medicine, Toyoake, Aichi, Japan; Genentech, UNITED STATES

## Abstract

**Objective:**

Pancreatic cancer is an aggressive tumor and the prognosis remains poor. Therefore, development of more effective therapy is needed. We previously reported that ^89^Zr-labeled TSP-A01, an antibody against transferrin receptor (TfR), is highly accumulated in a pancreatic cancer xenograft, but not in major normal organs. In the present study, we evaluated the efficacy of radioimmunotherapy (RIT) with ^90^Y-TSP-A01 in pancreatic cancer mouse models.

**Methods:**

TfR expression in pancreatic cancer cell lines (AsPC-1, BxPC-3, MIAPaCa-2) was evaluated by immunofluorescence staining. ^111^In-labeled anti-TfR antibodies (TSP-A01, TSP-A02) were evaluated *in vitro* by cell binding assay with the three cell lines and by competitive inhibition assay with MIAPaCa-2. *In vivo* biodistribution was evaluated in mice bearing BxPC-3 and MIAPaCa-2 xenografts. Tumor volumes of BxPC-3 and MIAPaCa-2 were sequentially measured after ^90^Y-TSP-A01 injection and histological analysis of tumors was conducted.

**Results:**

MIAPaCa-2 cells showed the highest TfR expression, followed by AsPC-1 and BxPC-3 cells. ^111^In-TSP-A01 and ^111^In-TSP-A02 bound specifically to the three cell lines according to TfR expression. The dissociation constants for TSP-A01, DOTA-TSP-A01, TSP-A02, and DOTA-TSP-A02 were 0.22, 0.28, 0.17, and 0.22 nM, respectively. ^111^In-TSP-A01 was highly accumulated in tumors, especially in MIAPaCa-2, but this was not true of ^111^In-TSP-A02. The absorbed dose for ^90^Y-TSP-A01 was estimated to be 8.3 Gy/MBq to BxPC-3 and 12.4 Gy/MBq to MIAPaCa-2. MIAPaCa-2 tumors treated with 3.7 MBq of ^90^Y-TSP-A01 had almost completely disappeared around 3 weeks after injection and regrowth was not observed. Growth of BxPC-3 tumors was inhibited by 3.7 MBq of ^90^Y-TSP-A01, but the tumor size was not reduced.

**Conclusion:**

^90^Y-TSP-A01 treatment achieved an almost complete response in MIAPaCa-2 tumors, whereas it merely inhibited the growth of BxPC-3 tumors. ^90^Y-TSP-A01 is a promising RIT agent for pancreatic cancer, although further investigation is necessary to improve the efficacy for the radioresistant types like BxPC-3.

## Introduction

Pancreatic cancer is one of the most aggressive tumors and the seventh leading cause of cancer death worldwide, accounting for 337,872 of the estimated new cancer cases and 330,372 of estimated cancer deaths (GLOBOCAN 2012, http://globocan.iarc.fr/). Since the symptoms of pancreatic cancer do not appear during its early stage and the majority of patients with the disease are already in an unresectable state at the time of diagnosis due to local invasion or metastatic spread [1–4]. The prognosis is very poor, namely, the 5-year survival rate for all staged disease is 6% [5]. It is projected to become the second leading cause of cancer death by 2030 in USA [5]. Therefore, additional effective anticancer therapy is necessary to augment and/or complement the present treatment strategies of surgery and chemo/radiotherapy, especially for patients with advanced pancreatic cancer.

Transferrin receptor (TfR), a type II transmembrane glycoprotein found as a homodimer (180 kDa) on the surface of cells, is involved in iron uptake through interaction with transferrin, and also in the regulation of cell growth [6,7]. Although TfR is expressed at low levels on normal cells, it is expressed at higher levels on cells with high proliferation rates, such as cancer cells [8–11]. TfR is therefore an attractive molecule for targeted therapy of cancer since its expression is upregulated on the cell surface of many cancer types including pancreatic cancer [10,12,13].

We previously reported that a ^89^Zr-labeled anti-TfR antibody (TSP-A01) is highly accumulated in the TfR-expressing tumor, MIAPaCa-2, derived from human pancreatic cancer, whereas its accumulation was low in the major normal organs [14]. TSP-A01 therefore has the potential to be used for radioimmunotherapy (RIT) by substituting positron-emitting Zr-89 with β- or α-emitting radionuclides with the appropriate physical properties. The concept of RIT has been applied in clinics for the treatment of non-Hodgkin B cell lymphoma, in which anti-CD20 antibody labeled with Y-90 or I-131 has been used [15]. RIT for solid tumors has not been approved by regulatory authorities for treating cancer to date. Y-90 is a pure β-emitter with a high energy level (maximum energy, 2.3 MeV) and an appropriate half-life (64.1 h) for RIT with IgG [16]. TSP-A01 internalizes into cells after binding TfR [14], and radiometal such as Y-90, which is expected to retain inside cells after internalization [15], is suitable for RIT with TSP-A01. In the present study, we evaluated and compared the *in vitro* and *in vivo* properties of two radiolabeled fully human anti-TfR monoclonal antibodies, TSP-A01 and TSP-A02, to determine the most suitable antibody for experimental RIT. The efficacy of ^90^Y-labeled TSP-A01 was then evaluated in mice bearing pancreatic cancer xenografts and compared with the efficacy of external beam radiotherapy (EBRT) with X-rays for the same tumor mouse models.

## Materials and Methods

### Cells

Human pancreatic cancer cell lines (AsPC-1, BxPC-3, and MIAPaCa-2) were obtained from American Type Culture Collection (Manassas, VA, USA). MIAPaCa-2 cells were maintained in D-MEM medium (Sigma, St. Louis, MO, USA) supplemented with 5% fetal bovine serum (Sigma) in a humidified incubator maintained at 37°C with 5% CO_2_. AsPC-1 and BxPC-3 cells were maintained in RPMI 1640 medium (Wako Pure Chemical Industries, Osaka, Japan) supplemented with 10% fetal bovine serum.

### TfR protein expression analysis by immunofluorescence staining

Cells were grown on glass coverslips overnight and fixed in cold methanol for 5 min. Nonspecific binding of antibodies was blocked by applying a Block Ace reagent (Dainippon Pharmaceutical, Osaka, Japan) with 10% goat serum for 30 min. Cells were incubated with TSP-A01 as a primary antibody overnight at 4°C. A secondary anti-human antibody conjugated with Cy3 (Jackson Immuno Research Laboratories, West Grove, PA, USA) was applied for 30 min at room temperature. Nuclei were stained with DAPI in mounting medium (Vector Laboratories, Burlingame, CA, USA). The images were obtained with an exposure time of 2/3 sec for detecting TfR using a fluorescence microscope (Olympus, Tokyo, Japan). Intensity of staining was measured by image J (National Institutes of Health, Bethesda, MD, USA).

### Radiolabeling

The antibodies were conjugated with *p*-SCN-Bn-DOTA (DOTA) (Macrosyclics, Dallas, TX, USA) according to the previously described procedure [17] with slight modifications. Briefly, during gentle shaking, a three or five molar excess of *p*-SCN-Bn-DOTA in 13.7 μL DMSO was added to the antibodies (10 mg in 1 mL 0.05 M bicine-NaOH, 150 mM NaCl, pH 8.5), and incubated for 17 h at 25°C. Non-conjugated chelate was removed by size exclusion chromatography using a PD10 column (GE Healthcare Life Science, Little Chalfont, UK) and 0.1 M acetate buffer (pH 6.0) as the eluent. The conjugation ratio of DOTA to TSP-A01 and TSP-A02 was estimated to be approximately 2.0 and 3.5, respectively, by MALDI-TOF mass spectrometry. For In-111 labeling, 48 μg of DOTA-conjugated antibodies were mixed with 1.48 MBq of ^111^InCl_3_ in 0.5 M acetate buffer (pH 6.0) and the mixture was incubated for 30 min at room temperature. For Y-90 labeling, 300 and 1000 μg of DOTA-conjugated TSP-A01 were mixed with 133.2 and 222.0 MBq of ^90^YCl_3_ in 0.5 M acetate buffer (pH 6.0), respectively, and the mixture was incubated for 30 min at room temperature. Radiolabeled antibodies were separated from free In-111 or Y-90 by Sephadex G-50 column (700×g for 2 min once or twice). The labeling yields of ^111^In-labeled TSP-A01 and TSP-A02 were approximately 50% to 90%. The labeling yields of ^90^Y-TSP-A01 were approximately 40% and 80%. The radiochemical purities of all labeled antibodies exceeded 97%. The specific activities of ^111^In-TSP-A01 were 17.8 to 26.9 kBq/μg, those of ^111^In-TSP-A02 were 15.6 to 28.7 kBq/μg, and those of ^90^Y-TSP-A01 were 173.7 and 178.5 kBq/μg.

### *In vitro* assays

Cell binding and competitive inhibition assays were conducted as previously described [18]. Briefly, in the cell binding assays, 3–4 days after seeding, we detached cells and prepared cell suspension. AsPC-1, BxPC-3, and MIAPaCa-2 (5.0 × 10^6^, 2.6 × 10^6^, 1.3 × 10^6^, 6.3 × 10^5^, 3.1 × 10^5^, 1.6 × 10^5^, 7.8 × 10^4^, and 3.9 × 10^4^) cells in PBS with 1% BSA (Sigma) were incubated with ^111^In-labeled antibodies (925 MBq) on ice for 60 min. After washing, the radioactivity bound to cells was measured. Immunoreactivity of ^111^In-labeled antibodies was estimated according to the method of Lindmo *et al* [19]. Cell binding data of ^111^In-labeled antibodies were analyzed by ANOVA, and the correlation between cell binding and TfR expression was examined by simple regression analysis. In competitive inhibition assays, 3–4 days after seeding, we detached cells and prepared cell suspension. ^111^In-labeled antibodies (925 MBq) were incubated with MIAPaCa-2 (2.0 × 10^6^) cells in the presence of varying concentrations of the corresponding unlabeled intact or DOTA-conjugated antibodies (0, 0.3, 0.6, 3.0, 6.1, 30.3, and 60.6 nmol/L) on ice for 60 min. After washing, the radioactivity bound to cells was counted. The dissociation constant (K_d_) was estimated using GraphPad Prism software (GraphPad Software, La Jolla, CA, USA).

### Subcutaneous tumor mouse models

The animal experimental protocol was approved by the Animal Care and Use Committee of the National Institute of Radiological Sciences, and all animal experiments were conducted in accordance with the institutional guidelines regarding animal care and handling. BALB/c-nu/nu male mice (5 weeks old, Japan SLC, Shizuoka, Japan) were maintained under a specific pathogen-free condition. For biodistribution experiments, mice were inoculated subcutaneously with BxPC-3 (4 × 10^6^) and MIAPaCa-2 (1 × 10^7^) cells in the left and right thighs, respectively, under anesthesia with 1.5% isoflurane. For RIT and EBRT experiments, mice were inoculated subcutaneously with BxPC-3 or MIAPaCa-2 cells in the left thigh.

### Biodistribution of ^111^In-labeled antibodies

When the subcutaneous tumors reached a diameter of approximately 8 mm (5–6 weeks after inoculation for BxPC-3, 8–9 weeks for MIAPaCa-2), mice (n = 5 at each time point) were intravenously injected with 37 kBq of ^111^In-TSP-A01 or ^111^In-TSP-A02. The total injected protein dose was adjusted to 5 μg per mouse by adding the corresponding intact antibody. At 1, 2, 4, 7, and 10 days after the injection of ^111^In-labeled antibody, mice were euthanized by isoflurane inhalation, and blood was obtained from the heart. The tumor and major organs were removed and weighed, and radioactivity counts were measured using a gamma counter (Aloka, Tokyo, Japan). The data were expressed as the percentage of injected dose per gram of tissue (% ID/g) normalized to a 20-g body weight mouse. Tumor-absorbed doses for ^90^Y-labeled antibodies were estimated from the biodistribution data of ^111^In-labeled corresponding antibodies and the mean energy emitted per transition of Y-90, 1.495 × 10^–13^ Gy kg (Bq s)^-1^ [20], as described previously [21].

### RIT with ^90^Y-TSP-A01 and EBRT with X-rays

When the subcutaneous tumors reached a diameter of approximately 8 mm (5–6 weeks after inoculation for BxPC-3, 8–9 weeks for MIAPaCa-2), RIT and EBRT experiments were conducted. Mice (n = 5 for each dose) bearing a subcutaneous tumor (BxPC-3 or MIAPaCa-2) were injected with 0.74, 1.85, and 3.7 MBq of ^90^Y-TSP-A01 into a tail vein. Protein dose was adjusted to 25 μg for each preparation by adding the intact antibody. As a negative control (0 MBq), five mice were intravenously injected with intact TSP-A01 (25 μg protein/mouse). As a separate experiment, tumors (n = 5 each dose) were irradiated with 0, 15, 30, and 60 Gy of X-rays at a rate of 4.4 Gy/min with a TITAN-320 X-ray generator (Shimadzu, Kyoto, Japan). Other parts of the mouse body were covered with a brass shield to limit unnecessary radiation exposure. The body weight and tumor size were measured at least twice a week for 42 to 49 days. However, when the tumor reached 15 mm in diameter, the mouse was euthanized humanely by isoflurane inhalation. Tumor volume (mm^3^) was calculated as (length × width^**2**^)/2. The tumor volume data were analyzed by two-way repeated measures ANOVA.

### Histological analysis of tumors

As a separate experiment, tumor samples (n = 2 at each time point) were extirpated at days 1, 3 and 7 after 3.7 MBq of ^90^Y-TSP-A01 injection and fixed in 10% (v/v) neutral buffered formalin and embedded in paraffin for sectioning. Untreated tumors were used as a control. Sections (3-μm thick) were stained with hematoxylin and eosin (H&E). Apoptotic cells in tumors were stained by terminal deoxynucleotidyl transferase-mediated deoxyuridine triphosphate nick-end labeling (TUNEL) staining with a DeadEnd Colorimetric TUNEL system (Promega, Madison, WI, USA). Ki-67 antigen was detected using an anti-human Ki-67 polyclonal antibody (Dako Denmark, Glostrup, Denmark) as described previously [22]. Untreated tumor sections were also stained with Masson's trichrome and an anti-CD31 antibody (ab28364, Abcam, Cambridge, UK). CD31 immunostaining was conducted in the same manner as the Ki-67 staining, except for primary antibody dilution (1:50 for the anti-CD31 antibody). TUNEL- and Ki-67-positive cells were quantified by counting five randomly selected fields of each section at 400× magnification.

## Results

### TfR protein expression and *in vitro* characterization of ^111^In-labeled antibodies

**Fig 1:**
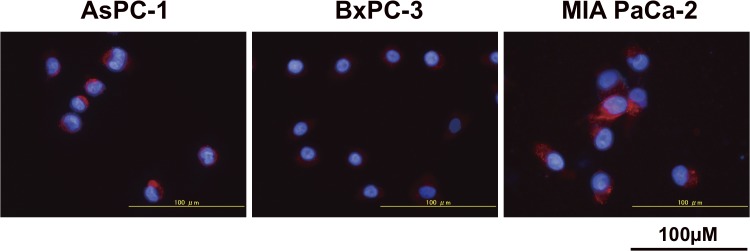
TfR protein expression analysis. The expression in pancreatic cell lines (AsPC-1, BxPC-3, and MIAPaCa-2) was determined by immunofluorescence staining with the anti-TfR antibody (red). DAPI stained nuclei (blue).

**Fig 2:**
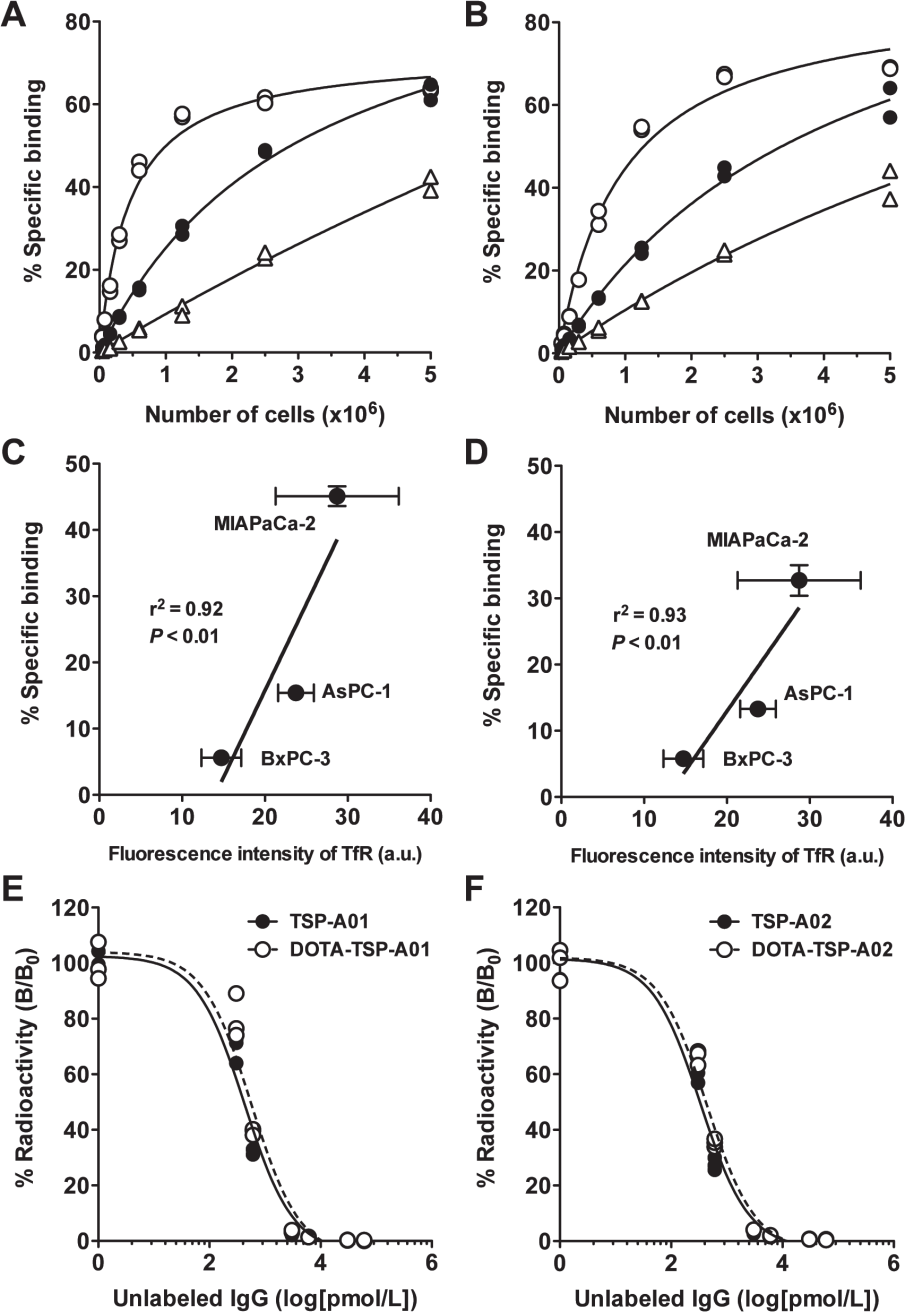
*In vitro* characterization of ^111^In-TSP-A01 and ^111^In-TSP-A02. Cell binding assay of ^111^In-TSP-A01 (A) and ^111^In-TSP-A02 (B) to AsPC-1 (black circles), BxPC-3 (white triangles), and MIAPaCa-2 cells (white circles). Correlation analysis of cell binding at 6.25 × 10^5^ cells with TfR protein expression for ^111^In-TSP-A01 (**C**) and ^111^In-TSP-A02 (**D**). The a.u. means arbitrary unit. Competitive inhibition assay for TSP-A01 (**E**) and TSP-A02 (**F**) using the corresponding intact (black circles, solid line) and DOTA-conjugated (white circles, dashed line) antibodies.

TfR protein expression of the three human pancreatic cancer cell lines (AsPC-1, BxPC-3, and MIAPaCa-2) determined by immunofluorescence staining with TSP-A01 indicated that MIAPaCa-2 showed the highest expression, followed by AsPC-1 and BxPC-3 (Fig 1). No signals were observed by immunofluorescence staining with the nonspecific antibody (S3 Fig). This was consistent with the observation in a murine cell line without human TfR expression [14]. In the cell binding assay for ^111^In-TSP-A01 (Fig 2A) and ^111^In-TSP-A02 (Fig 2B) with AsPC-1, BxPC-3, and MIAPaCa-2 cells; both ^111^In-labeled anti-TfR antibodies showed the highest binding to MIAPaCa-2 cells, followed by AsPC-1 and BxPC-3 (Fig 2A and 2B). The immunoreactive fraction of both ^111^In-labeled antibodies was estimated to be 1.0 based on the results of cell binding assays. The cell binding for both antibodies at 6.25 × 10^5^ cells was significantly correlated with TfR expression (*P* < 0.01) (Fig 2C and 2D). From the competitive inhibition assay, K_d_ of TSP-A01, DOTA-TSP-A01, TSP-A02, and DOTA-TSP-A02 was estimated to be 0.22, 0.28, 0.17, and 0.22 nM, respectively (Fig 2E and 2F). BxPC-3 and MIAPaCa-2 cells were selected as low and high expression models, respectively, for *in vivo* studies.

### Biodistribution of ^111^In-TSP-A01 and ^111^In-TSP-A02

**Fig 3:**
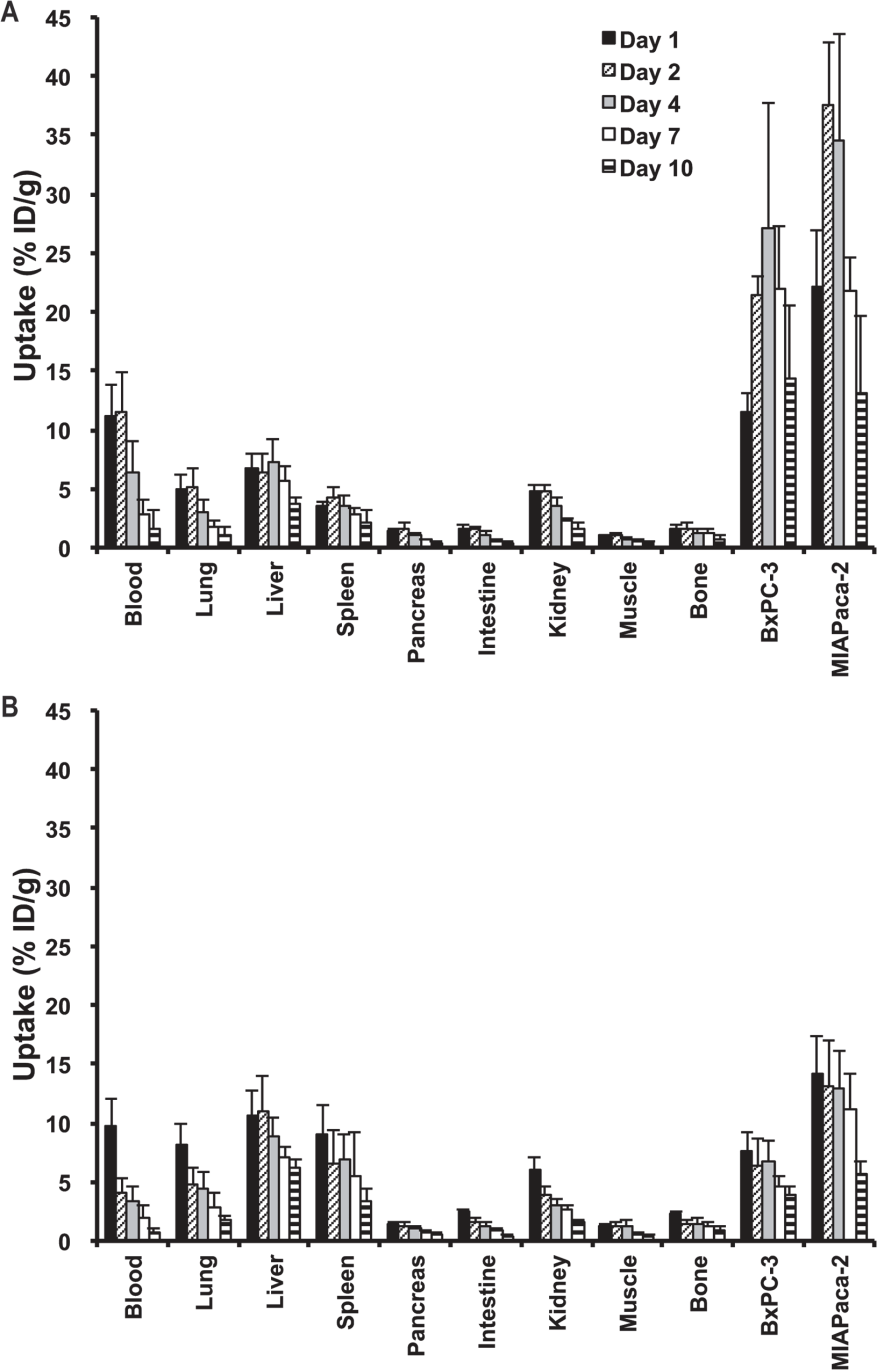
Biodistribution of ^111^In-TSP-A01 and ^111^In-TSP-A02 in nude mice bearing BxPC-3 and MIAPaCa-2 xenografts at day 1 (black bars), day 2 (diagonal line bars), day 4 (gray bars), day 7 (white bars), and day 10 (horizontal line bars) after intravenous injection of 37 kBq of ^111^In-TSP-A01 (A) and ^111^In-TSP-A02 (B).

Long-term biodistribution experiments of ^111^In-TSP-A01 (Fig 3A) and ^111^In-TSP-A02 (Fig 3B) were conducted in nude mice bearing both BxPC-3 and MIAPaCa-2 xenograft tumors from days 1 to 10 after injection. ^111^In-TSP-A01 accumulated in tumors at 11.5 ± 1.7% ID/g for BxPC-3 and 22.2 ± 4.8% ID/g for MIAPaCa-2 at day 1, with a peak value of 27.0 ± 10.7% ID/g at day 4 for BxPC-3 and 37.5 ± 5.3% ID/g at day 2 for MIAPaCa-2 (Fig 3A). Tumor uptake of ^111^In-TSP-A02 showed the peak value of 6.4 ± 2.4% ID/g for BxPC-3 and 13.1 ± 3.9% ID/g for MIAPaCa-2 at day 1 and decreased thereafter with time (Fig 3B). The peak of tumor uptake of ^111^In-TSP-A02 was significantly lower than that of ^111^In-TSP-A01 (*P* < 0.05 for BxPC-3, *P* < 0.01 for MIAPaCa-2), and the clearance of ^111^In-TSP-A02 from tumors was faster than that of ^111^In-TSP-A01 (Fig 3A and 3B). The uptake of both radiolabeled antibodies, especially that of ^111^In-TSP-A01, in the major normal organs was low (Fig 3A and 3B). The tumor-absorbed doses were estimated based on the biodistribution data of each corresponding ^111^In-labeled antibody. The dose absorbed by BxPC-3 tumors treated with 0.74, 1.85, and 3.7 MBq of ^90^Y-TSP-A01 was estimated to be 6.1, 15.4, and 30.7 Gy, respectively, and that by MIAPaCa-2 tumors was 8.9, 22.3, and 44.6 Gy, respectively. The dose absorbed by BxPC-3 tumors treated with 0.74, 1.85, and 3.7 MBq of ^90^Y-TSP-A02 was estimated to be 1.9, 4.8, and 9.7 Gy, respectively, and that by MIAPaCa-2 tumors was 3.8, 9.6, and 19.2 Gy, respectively. ^90^Y-TSP-A01 was therefore selected for the following RIT studies.

### RIT with ^90^Y-TSP-A01 and EBRT with X-rays

**Fig 4:**
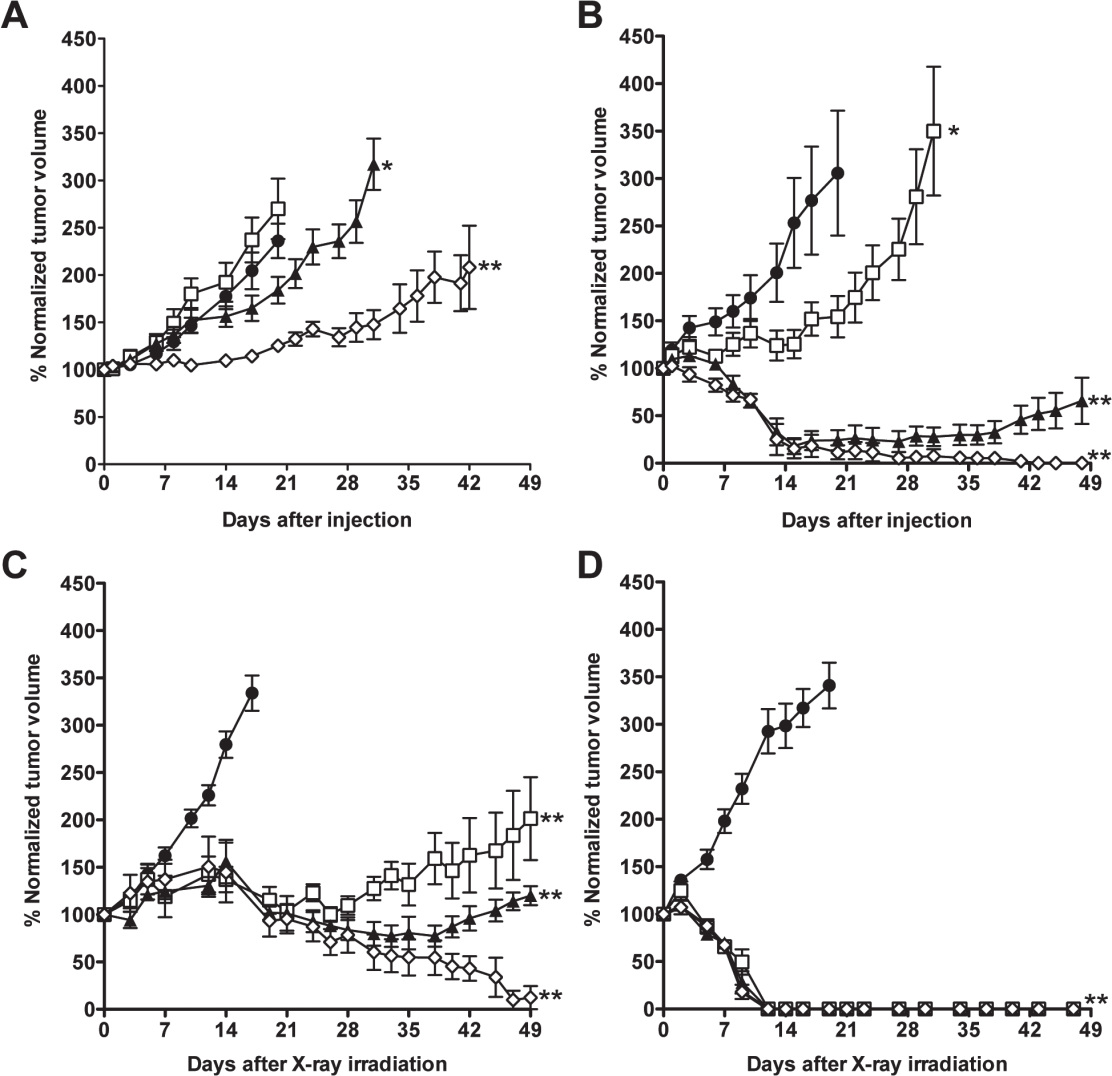
Growth curves of BxPC-3 **(A)** and MIAPaCa-2 **(B)** tumors in mice treated with 0 MBq (black circles), 0.74 MBq (white squares), 1.85 MBq (black triangles), and 3.7 MBq (white diamonds) of ^90^Y-TSP-A01. *P < 0.05, **P < 0.01 (vs. 0 MBq). Growth curves of BxPC-3 (**C**) and MIAPaCa-2 (**D**) tumors of mice treated with 0 Gy (black circles), 15 Gy (white squares), 30 Gy (black triangles), and 60 Gy (white diamonds) of X-rays. **P < 0.01 (vs. 0 Gy).

In BxPC-3 xenografts, RIT with 1.85 and 3.7 MBq of ^90^Y-TSP-A01 delayed tumor growth compared with the 0-MBq group and there were significant differences in tumor volume between 0-MBq and the two ^90^Y-TSP-A01 treatment groups (*P* < 0.05 for 1.85-MBq, *P* < 0.01 for 3.7-MBq) (Fig 4A). The 3.7-MBq treatment suppressed the BxPC-3 tumor growth until about 2 weeks after injection, and thereafter the tumor volume slowly increased (Fig 4A). Although tumor growth of the 0.74-MBq treatment group was a little faster than that of the 0-MBq group, the difference was not statistically significant (Fig 4A). It might reflect a minor difference in intrinsic tumor growth rates between the two groups, indicating that the 0.74-MBq treatment was not effective to BxPC-3 tumors. In MIAPaCa-2 xenografts, although tumor growth of the 0.74-MBq treatment group was delayed until around 2 weeks after injection, the tumor volume started to increase thereafter (Fig 4B). MIAPaCa-2 tumor volumes in 1.85- and 3.7-MBq treatment groups markedly decreased to approximately 20% around 2 weeks after injection. The tumors treated with 1.85 MBq of ^90^Y-TSP-A01 did not completely disappear and started to grow again about 6 weeks after injection, whereas all tumors of the 3.7-MBq treatment group had completely disappeared at 6 weeks after injection (Fig 4B). Although the body weight of almost all mice treated with ^90^Y-TSP-A01 temporarily decreased until around 7 days after injection, it increased thereafter and recovered around 2–3 weeks (S1 Fig). No severe adverse effects such as diarrhea were observed. Next, EBRT was conducted in the same tumor models as RIT (Fig 4C and 4D). Tumor volumes of BxPC-3 in all treatment groups increased until about 2 weeks after irradiation, but they decreased thereafter (Fig 4C). Regrowth of BxPC-3 tumors treated with 15 and 30 Gy was observed around 4 and 6 weeks after irradiation, respectively, whereas tumors treated with 60 Gy had nearly completely disappeared around 7 weeks after irradiation (Fig 4C). In MIAPaCa-2 xenografts, all tumors treated with radiation (15–60 Gy) showed a size decrease and had completely disappeared by about 2 weeks after irradiation (Fig 4D). Additional evaluation of *in vitro* radiosensitivity in BxPC-3 and MIAPaCa-2 cells also showed that BxPC-3 cells were more radioresistant compared with MIAPaCa-2 (S2 Fig).

### Histological analysis

**Fig 5:**
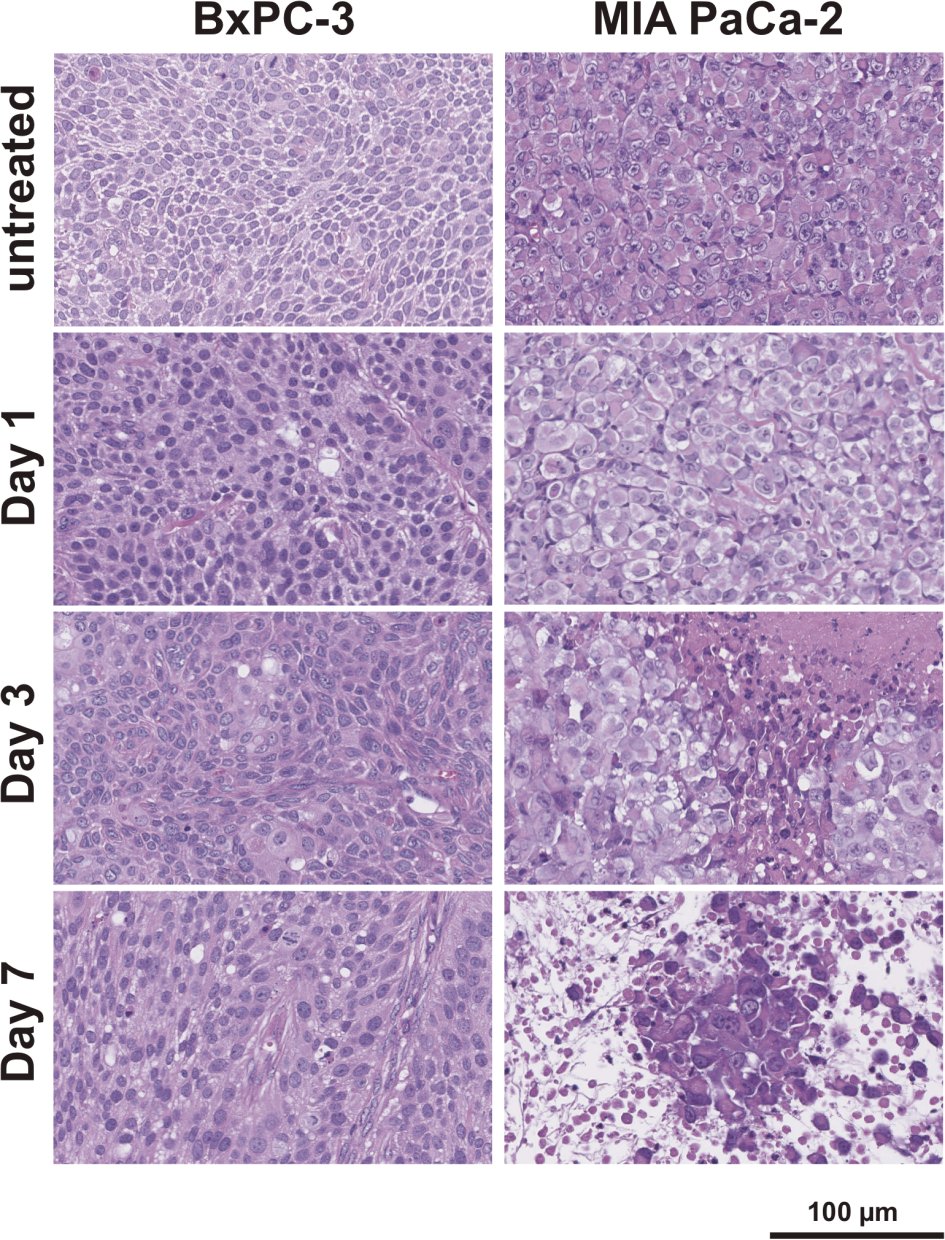
Histological analysis of tumors with ^90^Y-TSP-A01 treatment. H&E-stained BxPC-3 (left panels) and MIAPaCa-2 (right panels) tumor sections of untreated and at days 1, 3, and 7 after injection of 3.7-MBq ^90^Y-TSP-A01.

**Fig 6:**
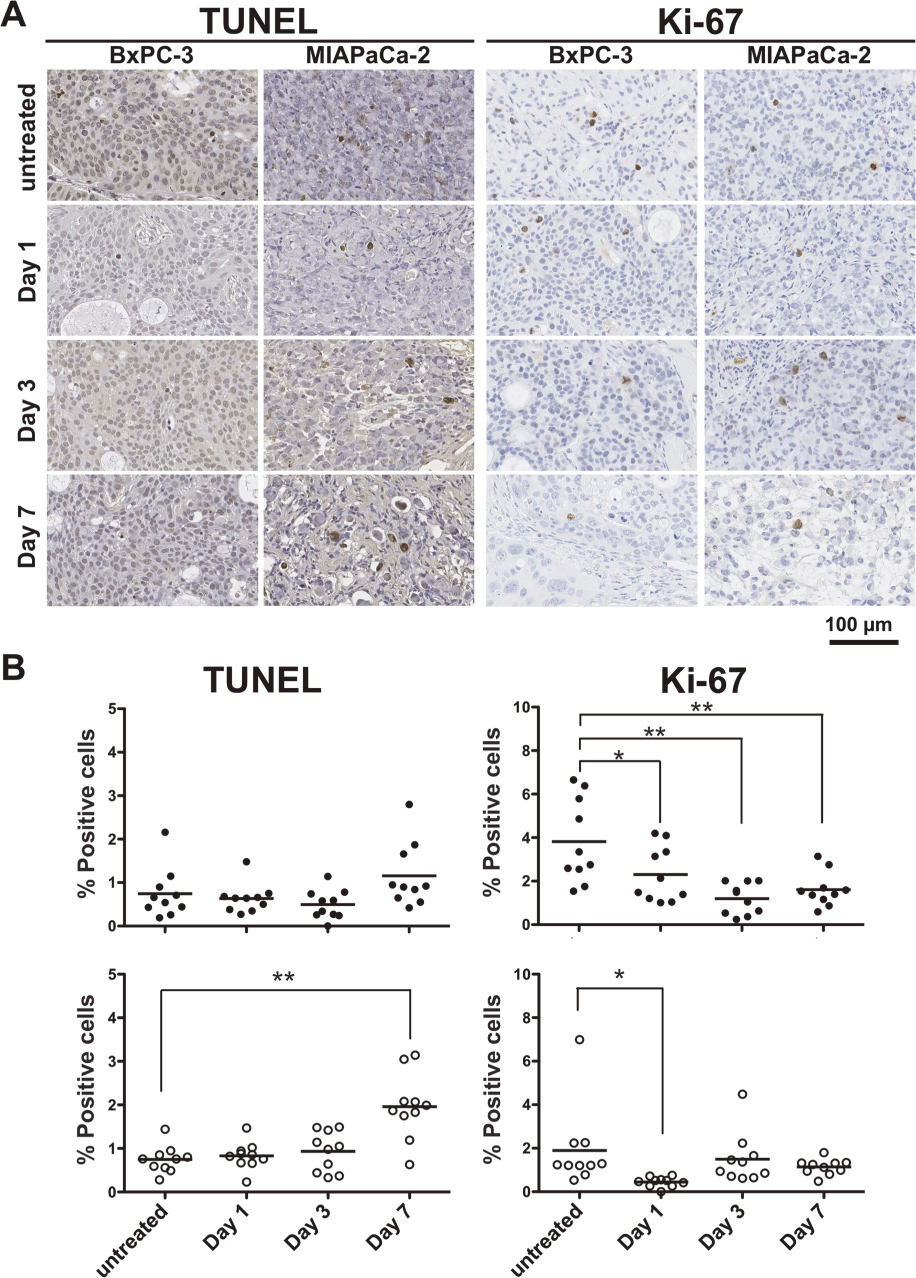
Immunohistological analysis of tumors with ^90^Y-TSP-A01 treatment. (**A**) TUNEL- (left side panels) and Ki-67-stained (right side panels) tumor sections at days 1, 3, and 7 after injection of 3.7-MBq ^90^Y-TSP-A01. (**B**) Scatter plots of positive cells in tumors treated with ^90^Y-TSP-A01 in BxPC-3 (black circles, upper panel) and MIAPaCa-2 tumors (white circles, lower panel). Bars indicate the mean. *P < 0.05, **P < 0.01 (vs. untreated).

**Fig 7:**
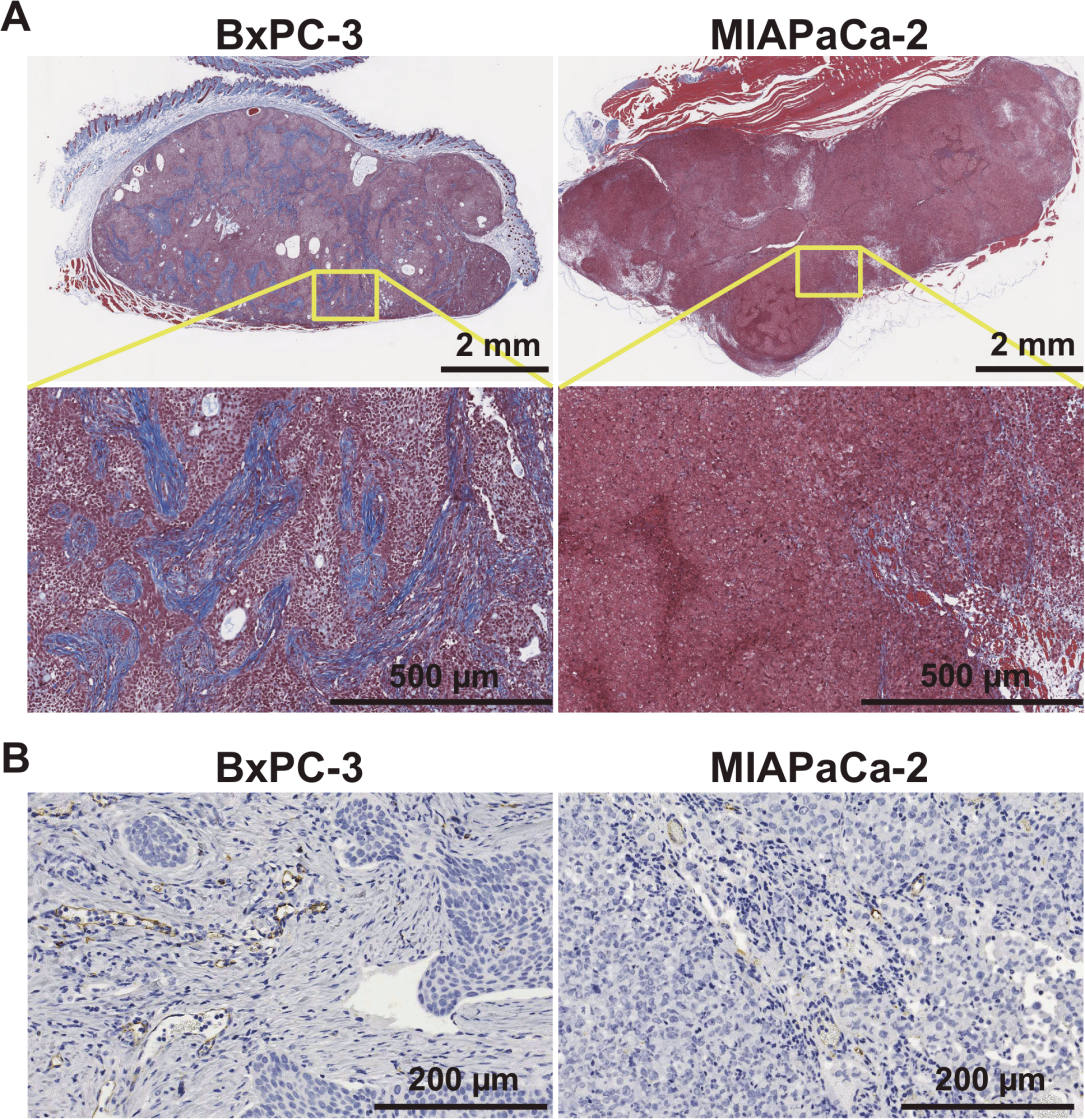
Masson's trichrome stained (A) and CD31-immunohistochemical stained (B) sections of untreated BxPC-3 (left panels) and MIAPaCa-2 (right panels) tumors.

Based on H&E-stained sections, in BxPC-3 tumors treated with 3.7 MBq of ^90^Y-TSP-A01, there was no significant difference compared with untreated tumors until 7 days after injection (Fig 5 left panels). In contrast, in MIAPaCa-2 tumors, cellular hypertrophy and necrosis were observed at day 3 after injection, and thereafter cell destruction and fibrosis were observed at day 7 (Fig 5 right panels). In TUNEL-stained sections, although apoptotic cells showed a tendency to increase at day 7 in treated BxPC-3 tumors (Fig 6A left side panels and 6B left panels), there was no significant difference in the percentage of apoptotic cells between RIT and untreated tumors (Fig 6B left upper panel). In contrast, in MIAPaCa-2 tumors there was a significant difference at day 7 between RIT and untreated tumors (*P* < 0.01, Fig 6B left lower panel). Ki-67-positive cells were decreased in BxPC-3 tumors by RIT and there were significant differences at days 1, 3, and 7 (*P* < 0.05 for day 1, *P* < 0.01 for days 3 and 7, Fig 6A right side panels and 6B right upper panel), whereas Ki-67-positive MIAPaCa-2 cells were significantly decreased only at day 1 after RIT (*P* < 0.05); there was no significant difference at days 3 and 7 (Fig 6A right side panels and B right lower panel). In Masson's trichrome-stained sections of untreated tumors, more abundant and thick stroma tissues were observed in BxPC-3 tumors compared with MIAPaCa-2 (Fig 7A). From CD31-stained sections of the untreated tumors, a few blood vessels were observed in both BxPC-3 and MIAPaCa-2 tumors (Fig 7B). Most of the vessels in BxPC-3 tumors were surrounded by abundant stromal tissues, but not in MIAPaCa-2 (Fig 7B).

## Discussion

Pancreatic cancer is an aggressive tumor form and the prognosis is very poor as the majority of patients present with metastatic disease mainly in the liver and peritoneal cavity, when they are diagnosed with pancreatic cancer [2,3]. Therefore, additional effective anticancer therapy is necessary for metastatic cancer. Pancreatic cancer cells express high levels of TfR, which are associated with increased cell growth and metastatic rates; TfR is therefore a key candidate molecule for diagnosis and therapeutics in pancreatic cancer [10,12,13,23,24]. We previously demonstrated that the radiolabeled anti-TfR antibody, ^89^Zr-TSP-A01, is highly accumulated in the pancreatic cancer xenograft, MIAPaCa-2, but not in major normal organs [14]. That finding suggests that RIT using TSP-A01 labeled with a cytotoxic radionuclide, such as Y-90, has the potential to be an effective treatment for pancreatic cancer.

In the present study, to select appropriate pancreatic tumor models, TfR protein expression analysis of three pancreatic cancer cell lines (AsPC-1, BxPC-3, and MIAPaCa-2) was conducted by immunofluorescence staining. The MIAPaCa-2 cell line showed the highest TfR expression and BxPC-3 showed the lowest. We screened human antibody libraries to obtained high affinity antibodies and isolated an additional antibody, TSP-A02, which had not been evaluated by biodistribution study. In the present study, TSP-A02 was employed in addition to TSP-A01 and the two antibodies were labeled with In-111 for evaluating *in vitro* and *in vivo* properties. The *in vitro* cell binding and competitive inhibition assays showed that the loss of immunoreactivity by radiolabeling procedures was limited and the affinity of TSP-A01 and TSP-A02 was nearly equivalent. ^111^In-TSP-A01 and ^111^In-TSP-A02 antibodies bound specifically to the three pancreatic cancer cell lines according to TfR expression, as determined by immunofluorescence staining. Therefore, MIAPaCa-2 and BxPC-3 cells were selected as high and low expression types, respectively, for the following *in vivo* evaluation.

To determine which antibody is suitable for RIT, the long-term biodistribution of the two ^111^In-labeled antibodies was evaluated in mice bearing both BxPC-3 and MIAPaCa-2 tumors, and the tumor-absorbed doses were estimated for the corresponding ^90^Y-labeled antibodies from the biodistribution data. Tumor uptake of ^111^In-TSP-A01 was markedly higher than that of ^111^In-TSP-A02 in both tumors, and estimated absorbed doses for ^90^Y-TSP-A01 by BxPC-3 and MIAPaCa-2 tumors were 3.2 and 2.3 times greater than those for ^90^Y-TSP-A02, respectively. In addition, ^111^In-TSP-A01 uptake in the major normal organs was lower compared with ^111^In-TSP-A02. TSP-A01 was therefore selected for the following RIT experiments.

TSP-A01 was labeled with Y-90 and evaluated in experimental RIT in mice bearing BxPC-3 and MIAPaCa-2 tumors. The therapeutic efficacy of ^90^Y-TSP-A01 in MIAPaCa-2 tumors was higher than that in BxPC-3, namely, the administration of 3.7-MBq ^90^Y-TSP-A01 decreased MIAPaCa-2 tumor size to near disappearance, but it merely delayed the growth of BxPC-3 tumors. From the histological analysis, ^90^Y-TSP-A01 RIT significantly induced apoptosis in MIAPaCa-2 tumors at day 7 after injection, but not in BxPC-3 tumors. ^90^Y-TSP-A01 RIT significantly suppressed cell proliferation in both tumors at day 1. At days 3 and 7, the proliferation level in MIAPaCa-2 tumors returned to approximately two-thirds the level of that in untreated tumors, whereas that in BxPC-3 was significantly lower and still less than 40% of that in untreated tumors. Radiation-induced apoptosis is known to occur following cell regrowth after transient cell cycle arrest [25]. The differing frequency of cell regrowth between BxPC-3 and MIAPaCa-2 tumors could lead to the difference in apoptosis induction.

The difference in the therapeutic efficacy of ^90^Y-TSP-A01 between BxPC-3 and MIAPaCa-2 tumors could be caused by several factors: 1) the TfR expression level in BxPC-3 cells is lower than that in MIAPaCa-2, resulting in less accumulation of ^90^Y-TSP-A01 in BxPC-3 tumors and a smaller tumor-absorbed dose in BxPC-3 tumors compared with MIAPaCa-2; 2) BxPC-3 tumor was more radioresistant compared with MIAPaCa-2, as confirmed by our *in vivo* EBRT experiments in tumor-bearing mice and the *in vitro* radiosensitivity assay in cultured cells; and 3) BxPC-3 has abundant cancer-associated stroma and most of the blood vessels are surrounded by the stromal tissues according to our histological experiments and also noted in several previous studies [26]. There are a number of parameters that influence the penetration of macromolecules, such as IgG, into the tumor tissues [27], and abundant cancer stroma surrounding the blood vessels could cause low penetration of macromolecules [27,28]. In our results of biodistribution experiments, ^111^In-TSP-A01 showed a lower accumulation in BxPC-3 tumors at the early time points compared with that in MIAPaCa-2, even though the tumor uptakes at the later time points were almost identical. The half-life of Y-90 is 64.1 h and its radioactivity exponentially decreases during the delayed accumulation in BxPC-3 tumors, therefore, it would lead to the lower absorbed doses in BxPC-3 tumors compared with those in MIAPaCa-2, especially at the early time points. A low initial dose of radiation is known to induce adaptive responses, sometimes inducing radioresistance to a subsequent high-dose irradiation [25]. The lower initial dose in BxPC-3 tumors might be a reason for the lower efficacy of RIT in BxPC-3 tumors compared with MIAPaCa-2 in addition to the intrinsic radioresistance of BxPC-3.

The results of RIT and EBRT experiments showed that the efficacy of RIT was markedly lower than that of EBRT with the corresponding radiation dose. Buras *et al*. reported that the efficacy of RIT was comparable to that of EBRT with γ-rays in a radiosensitive cell line, but not in a radioresistant cell line [29]. Although MIAPaCa-2 tumors were observed to be more radiosensitive than BxPC-3 in the present study, pancreatic cancer generally shows resistance to anti-cancer therapy including radiotherapy [30] and the lower efficacy of RIT with ^90^Y-TSP-A01 was observed in MIAPaCa-2 tumors compared with that of EBRT as well as in BxPC-3. MIAPaCa-2 cells may be more radioresistant compared with other types of tumor cells. Since it is generally difficult to treat wide-spread metastatic tumors with EBRT, RIT is expected to be one of the potential treatments for such tumors [31]. However, although ^90^Y-TSP-A01 was effective in MIAPaCa-2 tumors having high TfR expression and radiosensitivity, only a limited effect was obtained in BxPC-3 with a low TfR expression and radioresistance. Since this limited efficacy is a common problem with RIT for solid tumors [29,32], further investigation is necessary to determine the key factors associated with resistance to RIT and to improve the efficacy of RIT. BxPC-3 and ^90^Y-TSP-A01 could be a good model for such investigations. There are several potential strategies to improve the efficacy of RIT as follows: 1) combined therapy with a radiosensitizer such as gemcitabine [33–36], 2) radiolabeling with an α-emitter, such as Ac-225, which has more cytotoxicity than β-emitters [37], 3) improving antibody accessibility to tumor cells by decreasing cancer stroma [38], and 4) fractionated RIT, which can be given at a higher dose to tumors with manageable toxicity to normal organs compared with a single RIT infusion [39]. TSP-A01 is a fully human monoclonal antibody, therefore, it would be more suitable for fractionated RIT compared with murine and chimeric antibodies to avoid the human anti-mouse antibody response [40].

In conclusion, we evaluated the ^90^Y-labeled fully human anti-TfR antibody, TSP-A01, as a possible therapeutic application for pancreatic cancer in mouse models. ^90^Y-TSP-A01 was relatively effective in MIAPaCa-2 tumors having high TfR expression and radiosensitivity, but it was limited in BxPC-3 tumors showing low TfR expression and radioresistance. Although ^90^Y-TSP-A01 is a promising RIT agent, further study is necessary to improve the efficacy for clinical application to pancreatic cancer with radioresistance.

## Supporting Information

S1 Fig Body weight for mice bearing BxPC-3 (left) and MIAPaCa-2 (right) tumors treated with ^90^Y-TSP-A01 RIT. 0 MBq (black circles), 0.74 MBq (white squares), 1.85 MBq (black triangles), and 3.7 MBq (white diamonds) of ^90^Y-TSP-A01.
(EPS)

S2 Fig *In vitro* radiosensitivity assay. Cell survival curves in BxPC-3 (black circles) and MIAPaCa-2 (white circles) cells irradiated with 2, 4, 6, and 8 Gy of X-rays. Four days after irradiation, the cell survival was determined by a sulforhodamine B-based proliferation assay. **P < 0.01.
(EPS)

S3 Fig Immunofluorescence staining with nonspecific antibody. Three pancreatic cell lines (AsPC-1, BxPC-3, and MIAPaCa-2) were stained with the isotype control antibody (red). DAPI stained nuclei (blue).
(TIFF)

S1 Text Materials and methods for supporting information figures. (PDF)
