# Supplementary figures and images for: Evaluation of Efficacy of Radioimmunotherapy with 90Y-Labeled Fully Human Anti-Transferrin Receptor Monoclonal Antibody in Pancreatic Cancer Mouse Models
Source: PLoS One. 2015 Apr 20;10(4):e0123761. doi: 10.1371/journal.pone.0123761 (PMC4404254; doi:10.1371/journal.pone.0123761)

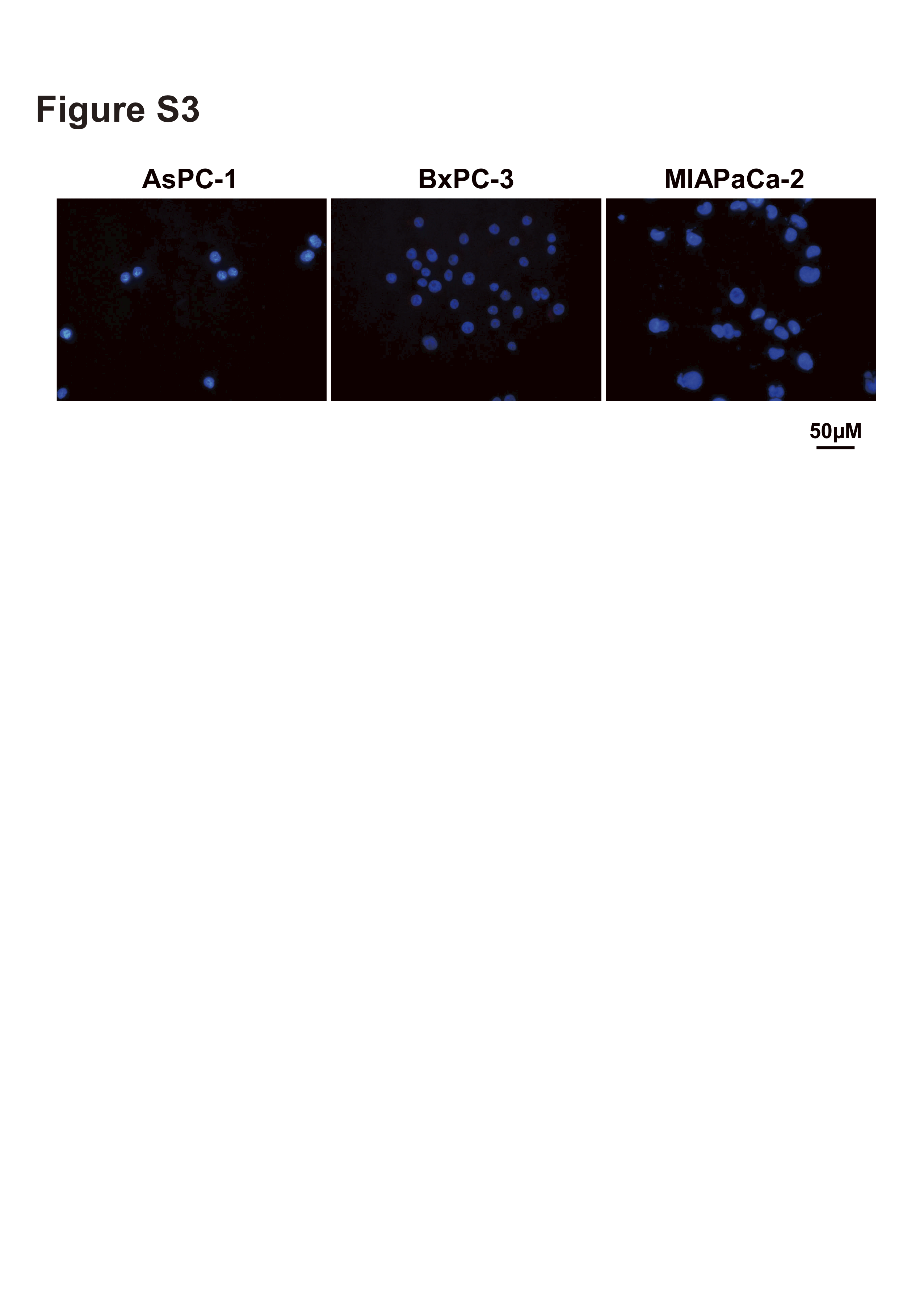

Supplement: S3 Fig — Three pancreatic cell lines (AsPC-1, BxPC-3, and MIAPaCa-2) were stained with the isotype control antibody (red). DAPI stained nuclei (blue). (TIFF) [file pone.0123761.s003.tiff]
