# Supplementary material for: Evaluation of Efficacy of Radioimmunotherapy with 90Y-Labeled Fully Human Anti-Transferrin Receptor Monoclonal Antibody in Pancreatic Cancer Mouse Models
Source: PLoS One. 2015 Apr 20;10(4):e0123761. doi: 10.1371/journal.pone.0123761 (PMC4404254; doi:10.1371/journal.pone.0123761)
Supplement: S1 Text — (PDF) [file pone.0123761.s004.pdf]

Supporting information Text S1. Materials and methods for supporting information figures.

Materials and methods for Figure S2

In vitro radiosensitivity assay was conducted as described previously [1]. Briefly, cell suspension of BxPC-3 and MIAPaCa-2 (5×10^4 cells/mL) was irradiated with 2, 4, 6, and 8 Gy of X-rays, and then BxPC-3 (3,000 cells/well) and MIAPaCa-2 (1,000 cells/well) cells were seeded in 96-well plates. Four days later, cell proliferation assay was conducted using a sulforhodamine B-based Toxicology Assay kit (Sigma, St. Louis, MO, USA). The surviving fraction was calculated as the ratio of absorbance of irradiated cells to that of non-irradiated cells and analyzed by two-way repeated measures ANOVA.

Materials and methods for Figure S3

Cells were grown on glass coverslips overnight and fixed in cold methanol for 5 min. Nonspecific binding of antibodies was blocked by applying a Block Ace reagent (Dainippon Pharmaceutical, Osaka, Japan) with 10% goat serum for 30 min. Cells were incubated with a primary antibody against SV40 large T antigen as an isotype control overnight at 4°C. A secondary anti-human antibody conjugated with Cy3 (Jackson Immuno Research Laboratories, West Grove, PA, USA) was applied for 30 min at room temperature. Nuclei

were stained with DAPI in mounting medium (Vector Laboratories, Burlingame, CA, USA). The images were obtained with an exposure time of 2/3 sec for detecting TfR using a fluorescence microscope (Olympus, Tokyo, Japan).

Supporting information Reference S1

1. Sudo H, Tsuji AB, Sugyo A, Ogawa Y, Sagara M, *et al*. (2012) ZDHHC8 knockdown enhances radiosensitivity and suppresses tumor growth in a mesothelioma mouse model. Cancer Sci 103: 203–209.
